# Socio-demographic factors and processes associated with stages of change for smoking cessation in pregnant versus non-pregnant women

**DOI:** 10.1186/1472-6874-11-3

**Published:** 2011-01-24

**Authors:** Alessandra Buja, Emanuela Guarnieri, Giovanni Forza, Federica Tognazzo, Paolo Sandonà, Alessandra Zampieron

**Affiliations:** 1 Department of Environmental Medicine and Public Health, Hygiene Institute, University of Padua, Italy; 2 Department of Gynecological Sciences and Human Reproduction, University of Padua, Italy; 3 Department of Environmental Medicine and Public Health, Toxicology Unit, University of Padua, Italy; 4 School of Nursing, University of Padua, Italy

## Abstract

**Background:**

The tobacco control community assumes that the most effective interventions are personalized. Nevertheless, little attention is paid to understanding differences between pregnant and non-pregnant European women in terms of the social factors that influence tobacco use and the processes of change used to quit smoking.

**Methods:**

The study consecutively enrolled 177 pregnant women who acknowledged smoking the year before pregnancy and 177 non-pregnant women who acknowledged smoking the year before their clinic visit for a Pap test.

**Results:**

With respect to socio-demographic factors, the stages of change in pregnant women were associated with level of education, marital status, and the presence of roommates, partners and friends who smoke. In pregnant women, there was no statistically significant difference in the processes used to stop smoking among the stages of change. Furthermore, behavioral processes were higher in non-pregnant women than in pregnant women, and the difference was statistically significant in the advanced stages of behavioral change. Both pregnant and non-pregnant women showed higher levels of acceptance towards smoking in the earlier stages of change, but the acceptability of smoking in the pre-contemplative stage was higher in non-pregnant women. Greater craving was detected in non-pregnant vs. pregnant women at all stages and reached a statistically significant level at the pre-contemplative stage.

**Conclusion:**

Pregnancy is a favorable time to stop smoking since pregnant women are more likely to be in an advanced stage of behavioral change. Pregnant and non-pregnant women are distinct populations in the types and processes of change involved in smoking cessation. The intervention programs to promote smoking cessation and prevent relapses will need to take these differences into account.

## Background

Despite the preventive measures that have been implemented by various governments resulting in a reduction of cigarette smoking [1], tobacco smoking is still a major cause of both fatal and non-fatal diseases [2,3] and one of the major causes of avoidable illnesses and premature death in Europe [4]. In particular, greater attention to public health prevention and intervention has been directed at women who smoke regularly because smoking rates among women, particularly girls and young women, are increasing in most developed and developing regions. Furthermore, some health risks related to smoking are unique to women (e.g., cervical cancer and lower infant birth weights), and other risks are higher in female smokers than their male counterparts (e.g., female smokers have their first heart attack at a younger age than male smokers) [5].

Among women, there are significant differences in smoking cessation and relapse between pregnant and non-pregnant smokers [6]. For example, the motivations to quit smoking are different in pregnant women compared with non-pregnant women. During pregnancy, many women improve their health habits with the goal of having a healthy baby [7,8]. In addition, the relapse rate in non-pregnant women is highest four weeks after the date they quit [9] whereas the highest rate of relapse among pregnant ex-smokers occurs in the postpartum period, often more than six months after quitting [10].

Undoubtedly, smoking cessation is a dynamic process with varying levels of motivation, intention, and confidence in quitting. The interventions for smoking cessation in non-pregnant women would be more effective if they could be stage- and process- specific, as described in the Transtheoretical Model (TTM) [11]. Nevertheless, while TTM-based interventions may have shown some evidence of a short-term benefit for quitting in pregnancy, there has been no benefit relative to standard care when followed-up in the longer-term [12]. The findings of Ruggiero et al. may explain this occurrence, as they found that pregnant women made less use of important experiential processes of change [13]; furthermore, Stotts and Scheibmeir found that pregnant women adopted less experiential and behavioral strategies to stop smoking [14-16]. Furthermore, much of the public health policy debate on smoking cessation has continued to focus on educational models of behavior change, which place individuals, rather than their environment, at the center of the debate [17]. In contrast, recent studies have identified the contextual, socio-environmental mechanisms that influence smoking behaviors and that probably differ for pregnant and non-pregnant women. Our review of the determinants of smoking cessation during pregnancy showed that factors such as socioeconomic status, education level, a partner's smoking habit and passive smoking may affect a woman's smoking behavior during pregnancy [18]. A cohort study of women verified that being married or in a committed relationship is significantly associated with quitting and that living in rural or remote areas and having lower educational attainment are associated with continued smoking [19].

To design the most effective interventions for these two distinct groups of women, we require more comprehensive information on how pregnant women who smoke differ from other women smokers across the different determinants of smoking cessation. These determinants include in the processes adopted in different stages of change, the situations that tempt women to smoke, the demographic and socio-environmental factors associated with the stages of change and the perceived acceptability of smoking. Thus, our study, conducted on a Mediterranean sample of pregnant and non-pregnant women smokers, aimed primarily 1) to assess the frequency of each stage of change in smoking cessation based on the Transtheoretical Model and 2) to examine the socio-demographic factors and processes associated with these stages for each group.

## Methods

### Setting

The study was performed at the Department of Gynecological Sciences and Human Reproduction, University Hospital of Padua, Veneto, for four months between December 2008 and March 2009.

### Sample

The study consecutively enrolled 177 pregnant women, smokers or ex-smokers, who acknowledged smoking nine months before pregnancy. The interview was performed by an obstetrician during the third trimester of pregnancy at pre-natal visits to the gynecology clinic. The study also consecutively enrolled 177 non-pregnant women, smokers or ex-smokers, who went to the same clinic for a gynecological Pap test screening and acknowledged smoking a year before the interview. The interview was performed by an obstetrician. The sample of both groups of women groups was drawn from the same waiting area of the same hospital during the same period. The inclusion criteria consisted of Italian-speaking women, without a diagnosis of cardiovascular or respiratory diseases, between 18 and 45 years old (fertile age). In addition, for pregnant women, a physiological pregnancy was required.

### Design

We conducted a quantitative research study with descriptive and analytical aims that had been authorized by the hospital and the department managers. Participants were informed about the aims of the study and provided consent. We adopted measures to safeguard privacy.

To apply the Transtheorical Model (TTM) of the stages of change, which were derived by a comparative analysis of theories on addiction (including smoking) [20-23], we used a questionnaire proposed by Prochaska et al. (1988) [24] that was translated into Italian by scientific translators and experts on addiction. "The Brief Version of the Processes of Change Questionnaire" derived from the TTM of change by Prochaska et al. [20] was administered; it included 20 items evaluated on a five-point Likert scale. This questionnaire evaluated the process of change as a personal mechanism that permits progression from one stage to another. It is based on five cognitive-experiential processes and five behavioral processes [21,24]. The experiential processes included consciousness raising, dramatic relief, environmental re-evaluation, social reappraisal and social liberation. In contrast, behavioral processes were comprised of stimulus control, helping relationships, counter-conditioning, reinforcement management and self liberation. All sample subjects (current and ex smokers) were asked about the age at which they initiated smoking and the number of cigarettes smoked one year ago; furthermore, current smokers were asked about the number of cigarettes smoked presently.

In addition, self-efficacy was evaluated on a scale designed by Velicer et al. [25] to measure people's attraction to smoking and the situations that increased their desire to smoke and thereby facilitated relapse. This questionnaire describes the situations that lead some people to smoke. The subjects have to describe how tempting it is to smoke in each situation. These situations are grouped based on three factors reflecting the most common types of tempting situations: negative or emotional distress, positive social situations, and craving.

Furthermore, we evaluated the social acceptability of smoking with an ad-hoc questionnaire based on a five-point Likert scale: "Not acceptable", "Slightly acceptable", "Moderately acceptable", "Acceptable", and "Very acceptable". The four items of this questionnaire investigated each woman's perception of the acceptability of smoking from different people's point of view: adult women, adolescent women, adult men, and the participant herself.

### Analysis

Sample characteristics were evaluated with a descriptive statistical approach. Inferential analysis was used to evaluate differences in frequency distribution in socio-demographic factors by stages of change. The chi-square test was applied when variables were categorical, and the exact Fisher test was used when expected frequencies were less than 5. To verify differences among the stages of change in average values of continuous quantitative variables with normal distributions, we applied the parametric test ANOVA. The Kruskal-Wallis and Mann-Whitney tests were used to evaluate differences when the quantitative variables were not normally distributed. The data were elaborated using Stata 8.1 software. The results of these analyses were reported as *p *values and considered statistically significant when p was less than 0.05.

## Results

### Sample Socio-demographic Characteristics

The mean age of pregnant and non-pregnant subjects was 32.6 ± 5.3 years and 34.8 ± 7.0 years, respectively, and 80.8% of pregnant women were married or common-law wives compared with 54.7% of non-pregnant women. In both groups, about 85% of the sample was Italian, employed, and had a high school education or above. Smoking was initiated at a mean age of 17.0 ± 2.9 years and 17.6 ± 2.6 years, respectively, for pregnant and non-pregnant subjects. There was no relevant difference detected in the mean age at which smoking was initiated between smokers and ex-smokers in both groups [Table 1]. In non-pregnant women, the mean number of cigarettes/day smoked one year ago for ex-smokers was 11.7 compared with 14.2 for current smokers; in pregnant women, the mean number of cigarettes/day was 10.1 in ex-smokers and 16.7 in current smokers. At the time of their interview, current smokers' mean number of cigarettes was 11.3 per day among non-pregnant women and 7.1 among pregnant women.

**Table 1:** Sample socio-demographic characteristics and stages of change by groups

**Sample characteristics**			
		**Pregnancy**	**Non Pregnancy**
Age (mean ± SD)		32.6 (± 5.3)	34.8(± 7.0)
Age at start smoking (mean ± SD)		17.0 (± 2.9)	17.6 (± 2.6)
Qualification (n, %)	Degree	65 (36.7%)	50 (29.1%)
	High School	77 (43.5%)	89 (51.7%)
	Mandatory education	35 (19.8%)	33 (19.2%)
Work (n, %)	Worker	22 (12.9%)	37 (21.5%)
	Employee	96 (56.1%)	88 (51.1%)
	Manager	28 (16.4%)	21 (12.2%)
	Unemployed	25 (14.6%)	26 (15.1%)
Marital status (n, %)	Married or common-law wife	143 (80.8%)	94 (54.7%)
	Not married	24 (13.6%)	49 (28.5%)
	Separated/Divorced	10 (5.6%)	29 (16.86%)
Nationality (n, %)	Italian	154 (87.0%)	147 (85.5%)
	Not Italian	25 (13.0%)	23( 14.5%)
Stage of change (n, %)	Pre-contemplative	18 (10.1%)	34 (19.8%)
	Contemplative	5 (2.8%)	36 (20.9%)
	In preparation	15 (8.5%)	22 (12.8%)
	In action	32 (18.1%)	39 (22.7%)
	In maintenance	107 (60.5%)	41 (23.8%)

### Stages of Change

The frequency distribution of women in the five stages of change was statistically different between pregnant vs. non-pregnant women (p < 0.01) [Table 1]. For the most part, pregnant women are in a stage of maintaining, whereas non-pregnant women were almost equally distributed among the five stages of change. The mean number of cigarettes smoked per day was 12.3 and 6.9 in pre-contemplative, 11.3 and 7.4 in contemplative, and 9.8 and 7.2 in preparation stages, for non-pregnant and pregnant women, respectively.

### Stages of change and socio-demographic variables

The analysis of the associations between socio-demographic variables and stages of change revealed that in pregnant women, the stages of change were associated with level of education (p = 0.03). Women with a college degree more frequently in the action stage, whereas women who attended only compulsory school were more often in a contemplative stage. Conversely, the stages of change in non-pregnant women were not associated with level of education. In pregnant women, marital status was also associated with the stages of change (p = 0.02). Married women or common-law wives were more often in the maintenance stage, whereas the stages of change in non-pregnant women were not associated with marital status. Furthermore, in pregnant women, we found an association between the stages of change and the presence of smokers living in the same house (p = 0.03), friends who smoke (p = 0.01), and smoking partners (p < 0.01). On the contrary, for non-pregnant women, living with smokers in the same house was the only variable associated with the stages of change (p = 0.01). No statistically significant association was found between employment and the stages of change for either group. Finally, there was no statistically significance association between the stages of change and either the women's ages or the age at which they began smoking.

### Processes of change

In non-pregnant women (Table 2), experiential processes were less active in the pre-contemplative group, while there were higher levels of behavioral processing among those in preparation and other advanced stages of change. However, in pregnant women, there were no significant differences in the processes used to stop smoking throughout the stages of change.

**Table 2:** Mean and standard deviation experiential and behavioural processes between stages of change in two groups

**Variabile**	**Pre-contemplative** **m(p50)**	**Contemplative** **m(p50)**	**In preparation** **m(p50)**	**In action** **m(p50)**	**In maintenance** **m(p50)**	**p Kruskal-** **Wallis test**
**Not pregnant**						
Experiential Processes	15.4 (3.1)	22.6 (6.2)	22.4 (4.7)	25.2 (4.6)	21.4 (4.0)	< 0.01
Behavioral processes	21.2 (6.0)	26.6 (7.5)	31.0 (6.3)	33.6 (7.1)	29.7 (9.2)	< 0.01
**Pregnant**						
Experiential Processes	19.4 (4.7)	21.1 (2.7)	22.6 (5.5)	21.4 (4.1)	20.1 (5.6)	0.40
Behavioral processes	20.7 (5.4)	25.0 (9.7)	25.5 (5.5)	23.8 (6.2)	20.8 (7.8)	0.05

**Figure 1:**
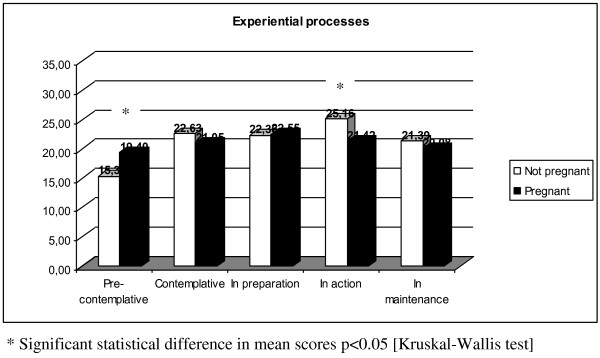
**Mean score of experiential processes between pregnant and not pregnant women**.

**Figure 2:**
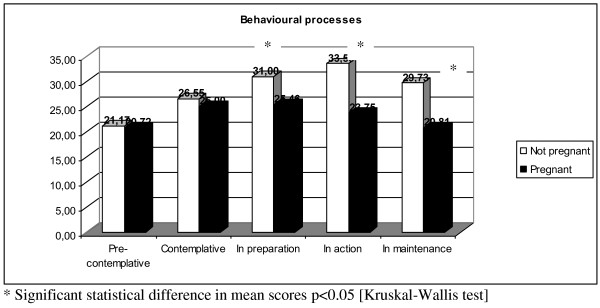
**Mean score of behavioural processes between pregnant and not pregnant women**.

The comparison of pregnant and non-pregnant women revealed a statistically significant difference in the mean scores of the experiential processes of those in the pre-contemplative stage (Figure 1). In contrast, behavioral processes were more active in non-pregnant women than pregnant women; this difference was statically significant among those in the advanced stages of change (Figure 2).

### The temptation to smoke

Table 3 shows a statistically significant association between the stages of change and three types of situations that encourage smoking (temptations): social setting, negative distress, and craving. Both pregnant and non-pregnant women in precocious stages experienced higher levels of temptation in situations where they felt social or emotional pressure. However, non-pregnant women struggled with more intense craving than their pregnant counterparts at all stages of change; this difference became statistically significant at the pre-contemplative stage. While there were generally no differences between the social and emotional temptations of pregnant and non-pregnant women as mentioned above, we did observe statistically significant lower temptation scores among pregnant women who were in a maintenance stage [data not shown].

**Table 3:** Mean and standard deviation of temptation score between stages of change in two women groups

**Variable**	**Pre-contemplative**	**Contemplative**	**In preparation**	**In action**	**In maintenance**	**p Kruskal-** **Wallis test**
**Not pregnant women**						
Social Situation	20.1 (± 5)	17.2 (± 5.3)	14.7 (± 5.7)	12.6 (± 5.9)	11.0 (± 4.6)	< 0.01
Negative Affect Situations	15.5 (± 2.5)	16.8 (± 2.7)	16.8 (± 3.3)	14.4 (± 4.0)	11.5 (± 4.2)	< 0.01
Craving Situation	10.8 (± 2.9)	10.1 (± 3.6)	9.2 (± 4.2)	6.6 (± 3.1)	5.6 (± 2.4)	< 0.01
**Pregnant women**						
Social Situation	18.7 (± 2.6)	20 (± 1.9)	15.6 (± 4.6)	13.8 (± 5.0)	10.1 (± 5.2)	< 0.01
Negative Affect Situations	16.7 (± 3.9)	17.2 (± 1.3)	15.7 (3.3)	12.7 (± 5.5)	8.8 (± 5.2)	< 0.01
Craving Situation	8.5 (± 2.6)	11.6 (± 1.7)	6.9 (± 3.1)	5.3 (± 2.6)	4.4 (± 2.2)	< 0.01

### The social acceptability of smoking

Both pregnant and non-pregnant women had higher levels of smoking acceptance in the early stages of change (p < 0.01) [Table 4]. A statistically significant difference in smoking acceptance was higher in non-pregnant women at the pre-contemplative stage, whereas there were no differences in scores during the latter stages between pregnant and non-pregnant women [data not shown].

**Table 4:** Mean and standard deviation of acceptability of smoke score between stages of change in two women groups

**Variabile**	**Pre-contemplative**	**Contemplative**	**In preparation**	**In action**	**In maintenance**	**p Kruskal-** **Wallis test**
	Not pregnant					
Acceptability of smoke	10,9 (± 4.4)	8.3 (± 3.2)	6.0 (± 2.4)	6.1 (± 2.6)	5.5 (± 2.5)	< 0.01
	Pregnant					
	8.3 (± 3.2)	6.2 (± 2.2)	7.5 (± 2.6)	6.7 (± 1.9)	6.1 (± 2.3)	< 0.01

## Discussion

This study depicts pregnant and non-pregnant women smokers as two distinct populations in several aspects. Behavioral process scores were higher in non-pregnant women in all stages; these differences were statistically significant in advanced stages of change. Furthermore, non-pregnant women experienced greater cravings than their pregnant counterparts in all stages; these differences were statistically significant in the pre-contemplative stage. Moreover, while both pregnant and non-pregnant women had higher levels of acceptance towards smoking in the pre-contemplative stage, non-pregnant women in this stage were more accepting of their smoking habit than those who were pregnant.

The present study also confirms a higher prevalence of non-pregnant women in earlier stages of change as compared with pregnant women. This result has been reported previously in earlier studies [26-28]. In fact, pregnancy provides a strong motivation to quit smoking based on a desire to give birth to a healthy baby and to be perceived as a responsible parent [29]. Our results and those of others seem to confirm that pregnancy is a favorable time to quit smoking.

Our results also reveal that the willpower to stop smoking in pregnant women is associated with certain socio-demographic factors, such as education, marital status, and living with or being around non-smokers (partners, friends, and colleagues). In the maintenance stage, there are a higher percentage of married women; conversely, in the pre-contemplative stage, there is a higher percentage of divorced or separated women. A pregnant woman in a stable relationship with a husband who does not smoke has more support to stop smoking [30]. Conversely, the stress of being pregnant without a partner or living with a husband who is a smoker makes a pregnant woman more inclined to smoke [31-35]. Furthermore, pregnant women with a higher level of education were more likely to be in the maintenance stage, suggesting that in pregnant women, education confers awareness of child health. However, education level does not seem to correlate with smoking among non-pregnant women. This finding is supported by Region Veneto data that evidenced no variation in behavioral risks such as smoking among different levels of education in non-pregnant women [36]. Furthermore a time-trend study conducted in northern Italy found that women with a low level of education who also exhibited low smoking levels were the only category to increase their smoking during the study, which narrowed the gap between them and more educated women in the sample [37]. The fact that educated women are accustomed to smoking and less inclined to quit smoking is a characteristic phenomenon of northern Italian culture that is probably is due to custom or to stress caused by high expectations and time pressures at work. No other socio-demographic factors promote smoking cessation in non-pregnant women besides living in a house with non-smokers.

In the pre-contemplative stage of change, both groups of women minimally utilize experiential and behavioral processes. As suggested by Prochaska [21], pre-contemplators process less information about smoking, spend less time re-evaluating themselves as smokers, and experience fewer emotional reactions to the negative aspects of smoking. However, the degree of experiential processing in non-pregnant women in the pre-contemplative stage was lower than that of pregnant women in the same stage. This suggests that even though a pregnant woman is pre-contemplative, it is likely that she has re-evaluated her environment due to her pregnancy and can be more easily "assimilated" into the contemplative group.

Pregnant women in all stages applied fewer behavioral processes, and the difference in scores reached statistical significance only in the advanced stages when behavioral processes are typically adopted to maintain smoking cessation. Pregnant women do not take actions that will reduce their cravings. It is possible that pregnancy itself strongly inhibits smoking, so behavioral processes are unnecessary to maintain smoking cessation during pregnancy. Ruggiero [13] stated that pregnant women are able to quit because of external factors and the belief that they only need to persist with their smoking cessation until the baby is born. However, pregnancy takes several months; after that, women have not activated the behavioral processes necessary to maintain their decision to stop smoking, and the experiential processes, including the awareness of health, likely wane after pregnancy. Prochaska demonstrated the importance of using different stages of change progressively [38]. Stotts [14] found that spontaneous quitters during pregnancy are more similar to non-pregnant women smokers who are in the contemplation or preparation stage of change before quitting than non-pregnant women who are in the process of quitting. These women who stop smoking during pregnancy seem to have suspended their smoking, rather than truly having quit. This lack of normal cessation coping activity may explain the high relapse rate in the first six months of the postpartum period, even though these women have gone without cigarettes for many months [39].

The Situational Temptation Measure [21] in both groups showed higher levels of temptation in each type of situation in the earlier stages of change; in both groups, reported temptation was higher in social situations than as the result of cravings. These findings agree with previously published studies [13]. Also, the acceptability of smoking decreased among the stages of change in both pregnant and non-pregnant women, with a higher level of acceptability in the pre-contemplative stage than in the other stages. These findings suggest that the desire to quit smoking in both pregnant and non-pregnant women arises from similar customs and social environments that influence these women's views as a function of their stage.

The major limitation of this study is the potential bias introduced by women's self-reporting of their smoking status; this bias is further enlarged by differential misclassifications in these two women's groups. In fact, a previous work dealing with this issue [40] found a that the reliance on self reported smoking status underestimated true smoking by 25% in pregnant women but not in non-pregnant women; in a Finnish study[41], the underestimation of current smoking in those participants who reported to have smoked at any time during their life, but not during the previous month, was 5.2% of women. However, it has been found that questionnaires administered by an interviewer yielded higher estimates of sensitivity and specificity than did self-administered questionnaires. Interviews more accurately identified smokers and classified non-smokers [42].

## Conclusions

In conclusion, pregnancy is a favorable time to quit smoking. Pregnant women are more likely to be in advanced stages of behavioral change, and socio-environmental-demographic factors significantly impact a woman's decision to quit smoking during pregnancy. It is very important that interventions potentiate and reinforce healthy environmental factors that can also affect perceptions of risk in pregnant women.

The processes of change are different for pregnant women than they are for non-pregnant women, suggesting the utility of different approaches in interventions for smoking cessation and relapse avoidance in the two groups. Effective interventions for pregnant women should be timed after the delivery or lactation period, encouraging the acquisition of behavioral processes and coping mechanisms that were not developed independently during pregnancy. For non-pregnant women, effective interventions to promote smoking cessation should focus on role-playing to address cravings, which are their most challenging hurdle.

## Competing interests

The authors declare that they have no competing interests.

## Financial Competing interests

The authors declare that they have no financial competing interests.

## Authors' contributions

FG, TF, AB and GE carried out the study design, the data collection and analysis and the manuscript preparation and critical revision. ZA participated in study design, manuscript preparation and critical revision. FG and TF helped to draft the manuscript. All authors read and approved the final manuscript.

## Pre-publication history

The pre-publication history for this paper can be accessed here:

http://www.biomedcentral.com/1472-6874/11/3/prepub
